# Investigation of vibrational manner of carbon nanotubes in the vicinity of ultrasonic argon flow using molecular dynamics simulation

**DOI:** 10.1038/s41598-021-96328-1

**Published:** 2021-08-19

**Authors:** Iman Karami, S. Ali Eftekhari, Davood Toghraie

**Affiliations:** Department of Mechanical Engineering, Khomeinishahr Branch, Islamic Azad University, Khomeinishahr, Iran

**Keywords:** Engineering, Nanoscience and technology

## Abstract

Among various types of nanostructures, carbon nanotube (CNT) is one of the most important nanostructures. These nanostructures have been considered due to their mechanical, thermal, and vibrational properties. In this research, this nanostructure’s vibrational behavior in the vicinity of argon flow in the vicinity of ultrasonic velocity was investigated. The effect of factors such as the stability of atomic structures, the atomic manner of carbon nanotubes in the presence of ultrasonic fluid, the influence of carbon nanotubes’ length, and the chirality of carbon nanotubes on vibrational behavior was studied by molecular dynamics simulation. The MD simulations display an enhance in amplitude and a decrease in the oscillation frequency. Physically, these simulations’ results indicated the appropriate mechanical strength of carbon nanotubes in the presence of argon fluid. Numerically, the simulated carbon nanotubes’ minimum oscillation amplitude and frequency were equal to 2.02 nm and 10.14 ps^−1^. On the other hand, the maximum physical quantities were expressed as 4.03 nm and 13.01 ps^−1^.

## Introduction

Nanotechnology is a field of applied knowledge that covers a wide range of industries in the world today. Nanotechnology is generally attributed to the science and technology of systems with scales less than 100 nm^1–5^. The most important attributes of carbon nanotubes are their mechanical and vibrational properties. Carbon nanotubes have a strength equivalent to 50 times that of steel, while their specific gravity is only one-sixth of steel, and they can withstand bending and tension without breaking. Carbon nanotubes have many applications and are used in various applications such as the construction industry, sports equipment, or reinforcement in composite materials. The research of Chwal^6^ is an essential source in the forthcoming simulations. Plimpton et al.^7^ performed experiments to obtain a final stress value of 11–63 GPa and the final strain of 10–13% for fifteen carbon nanotubes. They found that, on average, carbon nanotubes had final stress of 28 GPa and had good vibrational resistance to external stimuli. Using Molecular Dynamics (MD) simulation, Belytschko et al.^8^ expressed final strain, and final stress of carbon nanotubes with the chiral index of (12, 12) are equal to 14.3% and 97.5 GPa, respectively. Plimpton et al.^9^ investigated the elastic constants and predicted the stress–strain relationships for carbon nanotubes under torsional loads and tensile. They reported fluctuations in carbon nanotubes for different external loads. In a finite element model proposed by Marco Rossi et al.^10^, two single-walled carbon nanotubes’ mechanical and vibrational properties were calculated. They found that carbon nanotubes’ chirality played an important role in their failure and the number of oscillations. Tombler et al.^11^ reported the modulus of elasticity 1.2 TPa for single-walled carbon nanotubes using four different methods. Li et al.^12^ proposed a finite element model by replacing the beam element with a carbon–carbon bond. They showed that by changing the bond shape between carbon particles, the number of structural oscillations relatives to an applied external force increased. Ávila et al.^13^ calculated the modulus of elasticity of carbon nanotubes using the MD method. In connection with carbon nanotubes’ mechanical and vibrational behavior, Poelma et al.^14^ simulated carbon nanotubes by removing carbon particles. The obtained outcomes displayed that with enhancing the amount of these defects, the mechanical strength of carbon nanotubes decreased, and the amplitude and vibration frequency increased and decreased, respectively. Lu et al.^15^ analyzed the dynamic characteristics of CNT by the modified molecular structural mechanics method (MMSMM). In MMSMM, the deformation potential of CNT is decomposed. So the stiffness matrix of CNT is estimated. This algorithm computes the natural frequencies of CNT. Hu et al.^16^ present a review of vibrations of CNT investigated using the nonlocal rod model and nonlocal beam model, and molecular dynamics simulation. They show the nonlocal continuum models play an essential role in studying the vibration of CNTs. The applicability of the nonlocal continuum models based on the molecular dynamics simulation results is summarized in this work. Yoon et al.^17^ studied shear deformation and rotary inertia on transverse wave propagation in individual carbon nanotubes (CNTs). The outcomes are shown for transverse wave speeds of double-wall CNTs, based on the Euler-beam model and Timoshenko-beam model, respectively. The experimental observations show that the frequency value for CNT was about 10 THz. In our study, carbon nanotubes in various chiralities and sizes, along with argon fluid that flows at supersonic speed, are simulated with LAMMPS software. Its mechanical (vibrational) behavior is investigated. For this goal, simulated carbon nanotubes are considered ideal and without atomic defects. So, in current MD simulations, the atomic behavior of CNT structures in the vicinity of Ar atoms was reported for the first time. We expected that the computational outcomes improve the designing process of actual applications such as gas sensors, atomic membranes, etc.

## Computational methods

Molecular dynamics was one of the first simulation methods used to describe the dynamics of liquids by Alder and Wainwright^15^ and then by Rahman^16^ in the 1950s and 1960s. LAMMPS software is a simulation box according to classical physics and Newton’s laws. LAMMPS has abbreviated Large Scale Atomic/Molecular Massively Parallel Simulation^7,9,17,18^ (Also, See https://www.lammps.org). It is an open-source code, distributed freely under the terms of the GNU Public License (GPL) version 2. In MD simulation, the position and velocity of the particles can be calculated using the Newtonian equation of motion^15^. Newton’s equation of motion is solved using the velocity-Verlet algorithm^19^. Then, having the initial velocity and position of the atoms and considering the average temperature, the simulation begins. The most important part of molecular dynamics simulation is choosing the right potential function. Therefore, in this study, the potential function of Lennard–Jones, EAM, and Tersoff has been used to express the interaction of particles. The Lennard–Jones function is expressed as^20^ Eq. (1):

1 $$U_{LJ} = 4\varepsilon_{ij} \left[ {\left( {\frac{{\sigma_{ij} }}{{r_{ij} }}} \right)^{12} - \left( {\frac{{\sigma_{ij} }}{{r_{ij} }}} \right)^{6} } \right]\quad r < \, r_{c}$$

Here $\varepsilon_{ij}$ is the depth of the potential well and $\sigma_{ij}$ is the finite distance at which the potential function becomes zero, and $r_{ij}$ is the particles’ distance from each other. In the recent formulation, *r*_*c*_ is the cut-off radius in the simulations. The values $\varepsilon_{ij}$ and $\sigma_{ij}$ of the platinum, carbon, and argon particles are shown in Table 1.

**Table 1:** The parameters of Lennard–Jones potential function for different particles in MD simulations^20^.

Particle type	σ (Å)	ε (kcal/mol)
Ar	3.868	0.185
C	3.851	0.105
Pt	2.754	0.08

In addition to the Lennard–Jones interaction, the non-bonding potential function of EAM is used to express the interaction between metal particles according^21^ to Eq. (2):

2 $$U_{i} = F_{\alpha } \left( {\mathop \sum \nolimits_{i \ne j} \rho_{\beta } \left( {r_{ij} } \right) } \right) + \frac{1}{2} \mathop \sum \limits_{i \ne j} \phi_{\beta } \left( {r_{ij} } \right)$$

Here, F_α_ is a constant coefficient. ρ_β_ is a factor because of atomic charge density, and ϕ_β_ is a factor because of the presence of particles in the simulation box.

The particles interactions are described using the Tersoff potential and with the following formulation^22^:

3 $$U = { 1/2}\sum\limits_{i} {\sum\limits_{i \ne j} {V_{i} } }$$

4 $$U_{ij} = \, f_{c} \left( {r_{ij} } \right) \, \left[ {f_{R} \left( {r_{ij} } \right) \, + \, b_{ij} f_{A} \left( {r_{ij} } \right)} \right]$$

Here, the expression f_R_ shows repulsive interactions and f_A_ is the gravity type in simulated carbon systems.

In the simulations performed in this research, NVT and NVE ensembles are used. The thermostat is used to keep the temperature constant within a particular range^23,24^. In the present study, the Nose–Hoover thermostat is used to create thermal equilibrium in the simulated structures. Using the stated concepts of the MD approach, CNTs atomic behavior in the vicinity of Ar fluid can be described. In the current study, suitable boundary conditions for this mechanical process are periodic boundary conditions along x and y axes and fix one in the z-direction. From a dimensional point of view, the box length in current simulations is 75 * 75 * 150 Å, and the primary carbon nanotube used in the MD box has (7,7) chirality and 5 nm length. In terms of initial conditions, the initial temperature value of the simulated atomic structure is 300 K, which is applied using the Nose–Hoover thermostat.

**Figure 1:**
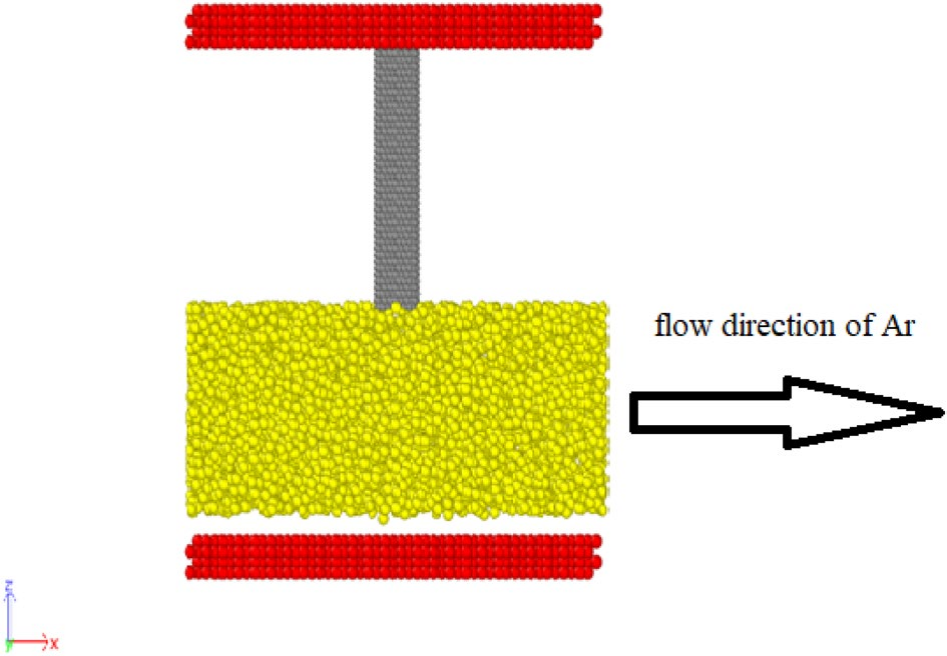
The schematic of a simulated carbon nanotube system and ultrasonic fluid (argon) which is created in LAMMPS^9,18,19^.

The time step in molecular dynamics simulations is another important and influential factor in the simulation results that the numerical value of this simulation constant is considered equal to Δt = 2 ns. In terms of temperature equilibrium, the fluid and carbon nanotubes in the simulation box have good stability and, the simulated system is balanced at the desired initial temperature. In this study, we investigate factors such as the stability of atomic structures, the atomic manner of carbon nanotubes in the presence of ultrasonic fluid, the effect of carbon nanotubes’ length, and the chirality of carbon nanotubes on vibrational manner. Numerically, the velocity of fluid particles in the MD simulation pakage is set to 400 m/s, and the frequency of atoms in this fluid is set to 25 kHz value. Argon fluid is selected as the base fluid in these simulations, and the arrangement of these particles is random. Finally, carbon nanotubes with the chiral index of (7, 7) are placed vertically at the top of the simulated channel. Simplification hypotheses are considered to simplify the simulation process. These hypothesis statements are: The present particles in the wall of carbon nanotubes are rigidly simulated. One end of the carbon nanotube is held in place. The temperature of the whole system is constant. The interaction between the fluid particles and the carbon nanotube does not change the wall structure of the carbon nanotube. An example of the atomic structure which is created in LAMMPS^9,18,19^ is shown in Fig. 1. In this figure, the top and bottom red color layers show the fluid flux duct limiting the fluid flux in a specified route, the yellow part is fluid and the gray part is the carbon nanotube.

## Discussion and results

### The stability of atomic structures

**Figure 2:**
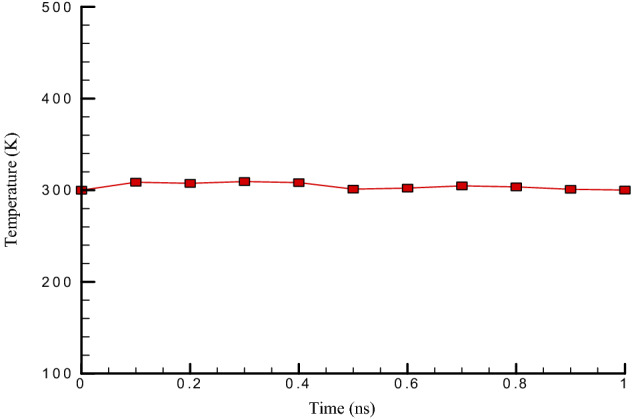
The Temperature changes vs. time in the existence of carbon nanotubes (7 and 7) with a length of 5 nm at the initial temperature (300 K).

**Figure 3:**
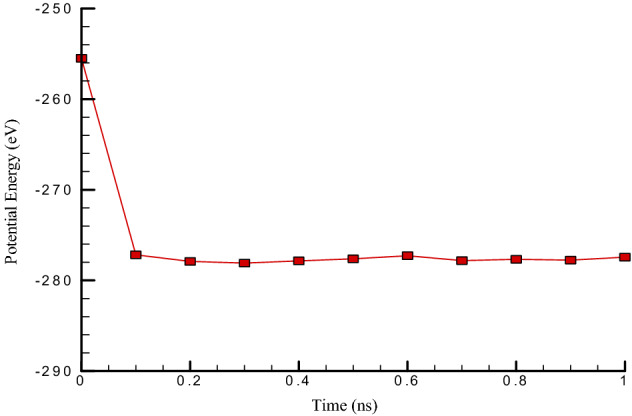
The Potential energy changes vs. time in the simulated atomic structure in the existence of carbon nanotubes (7 and 7) with a length of 5 nm at the initial temperature (300 K).

According to Figs. 2 and 3, it can be seen that the total temperature in the simulated atomic structure in this study is balanced at 300 K. Therefore, temperature fluctuations are not observed in it. These diagrams show that 1,000,000 time steps provide enough time to balance atomic structures. It can be said that with the proper selection of atomic positions and interactions between particles in simulated structures, the amount of temperature in these structures converges and the oscillations amplitude becomes smaller with increasing time steps so that this behavior, Fig. 2 is visible. Fig. 3 shows the potential energy changes over time in the simulated atomic structure. In terms of the potential energy of the simulated system, it can also be said that this structure is balanced. The physical quantity is negative and equal to − 227.43 eV. The potential energy in atomic structures is one of the important factors that can be used to study atomic structures’ stability. Physically, by negating the numerical value of potential energy in atomic structures, the force of gravity is established between the particles. As a result, the simulated structures have a thermodynamic equilibrium. Also, reducing the amplitude of fluctuations in the potential energy graph over time is another important factor in molecular dynamics studies. More precisely, reducing the oscillations amplitude in atomic structures indicates convergence, reducing motion, and atomic oscillations in the simulated structures.

**Figure 4:**
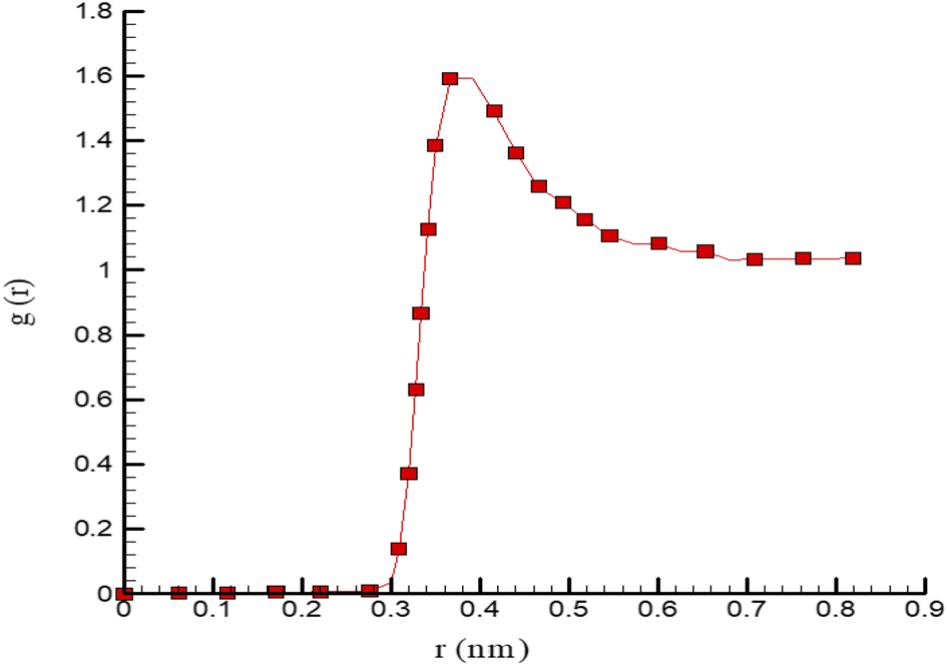
RDF curve of Ar fluid at T = 300 K as initial temperature.

Furthermore, we calculate the Radial Distribution Function (RDF) of Ar atoms. Our calculated results for this physical parameter are reported in Fig. 4. This figure shows that our MD study results are consistent with previous reports and display the validity of this simulation^13^. Technically, this validation process arises from 2 essential cases. First, our MD simulation settings, such as force-field definition and atomic modeling process, are done precisely. Secondary, MD time is sufficient to equilibrate atomic structures.

### The influence of carbon nanotube length on its vibrational behavior

**Figure 5:**
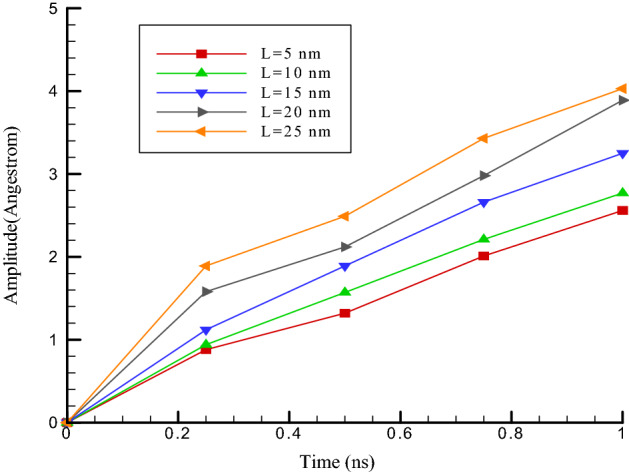
Oscillation amplitude of carbon nanotubes versus length of carbon nanotubes (7 and 7) in different time steps.

**Figure 6:**
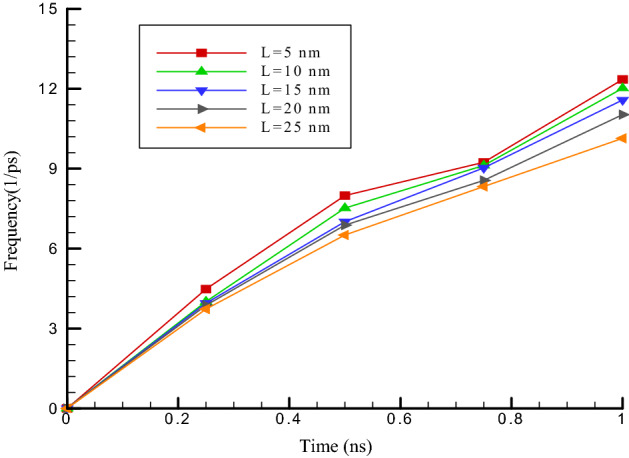
Oscillation frequency changes of carbon nanotubes versus length of carbon nanotubes (7 and 7) in different time steps.

This section investigates the influence of carbon nanotube length on its fluctuations in the vicinity of the ultrasonic fluid. The carbon nanotube length (7 and 7) is increased from 5 to 25 nm. To be more precise, the length of the carbon nanotubes in this section is equal to 5, 10, 15, 20, and 25 nm. The results of the simulations in this section show an increase in the amount of oscillation amplitude and a decrease in the oscillation frequency of the simulated structures so that by increasing the size of carbon nanotubes, the value of the oscillation amplitude arrives from 2.56 to 4.03 nm (see Fig. 5). Physically, the frequency in our MD simulations shows the number of occurrences of nanotube oscillation per unit of time. The amplitude and frequency of the simulated structure in the current work are calculated using “XYZ” out of the LAMMPS package. This output shows the position of atoms in each time step. So, we can estimate the maximum value of atomic displacement of CNT atoms structure. by this calculation the oscillation amplitude reported. Furthermore, number of timesteps between the two maximum displacements in the CNT structure will indicate the oscillation frequency. The outcomes display that increasing the length of nanocarbon from 5 to 25 nm reduces the Oscillation frequency from 12.35 to 10.14 ps^−1^ (see Fig. 6). Also, increasing the length of nanocarbon from 5 to 25 nm increases the value of the atomic interaction in the simulated structures from 56.31 to 69.75 eV. These changes due to the enhance in the number of particles interacting in the simulation pakage. The highest values of oscillation amplitude, oscillation frequency and interactive energy of carbon nanotubes (7 and 7) in terms of carbon nanotube length in the last step of simulation are reported in Table 2.

**Table 2:** Highest values of interactive energy, oscillation frequency and oscillation amplitude of carbon nanotubes (7 and 7) in terms of carbon nanotube length in the last step of molecular dynamics simulation.

Length of carbon nanotubes (nm)	Oscillation amplitude (nm)	Oscillation frequency (ps^−1^)	Interactive energy (eV)
5	2.56	12.35	56.31
10	2.77	12.02	59.59
15	3.25	11.58	61.25
20	3.89	11.03	65.51
25	4.03	10.14	69.75

**Figure 7:**
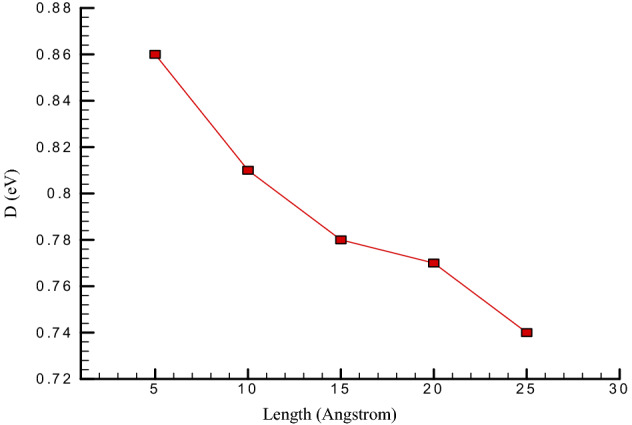
Bending Stiffness changes of carbon nanotubes versus length of nanotubes.

Stiffness is the extent to which an object resists deformation in response to an applied force. This physical parameter shows the mechanical stability of simulated structures. In the current work, we calculate the bending stiffness of CNT as a function of these nanotubes’ lengths by estimate of CNT atoms stress with “compute/STRESS” command in LAMMPS package. As depicted in Fig. 7, by CNT length enhancing from 5 nm to 25 nm, the stiffness of simulated structures decreases from 0.86 to 0.74 eV, respectively. This atomic behavior shows the mechanical weakening of CNT by length increasing.

### The chirality influence of carbon nanotubes on their vibrational behavior

The number of particles in carbon nanotubes increases by increasing chirality in these structures. As a result, the oscillating behavior of carbon nanotubes near argon is expected to increase. In this part of molecular dynamics simulations, the amount of chirality in carbon nanotubes structure is considered equal to (5, 5), (6, 6), (7, 7), (8, 8), and (9, 9). From the atomic point of view, with enhancing chirality in carbon nanotubes’ structure with a length of 5 nm, the diameter of carbon nanotubes enhances. As a result, it can be said that with enhancing chirality in atomic structures, the number of carbon particles in carbon nanotubes enhances, and as a result, the interactions of carbon nanotubes with ultrasonic fluid increase. The change in the chirality of carbon nanotubes has caused a change in its dimensions, and it affects the use of these atomic structures as oscillators. So with the increasing chirality of carbon nanotubes, the radius in this structure increases. Numerically, the amount of oscillation amplitude varies from 2.97 to 2.02 nm, and the atomic frequency value varies between 12.95 to 11.79 ps^−1^.

**Figure 8:**
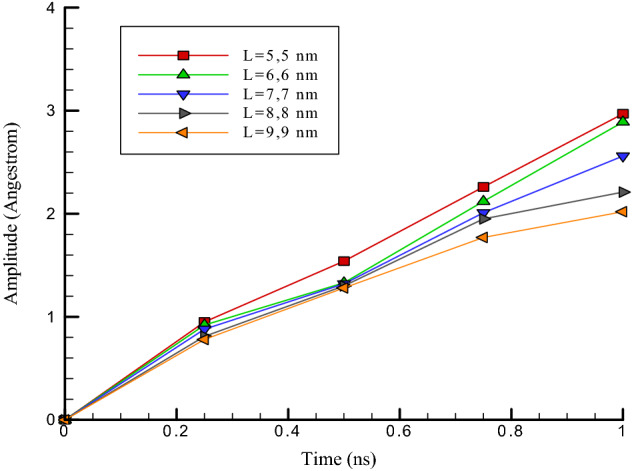
Oscillation amplitude changes of carbon nanotubes versus the frequency with a length of 5 nm in variuos chiralities.

**Figure 9:**
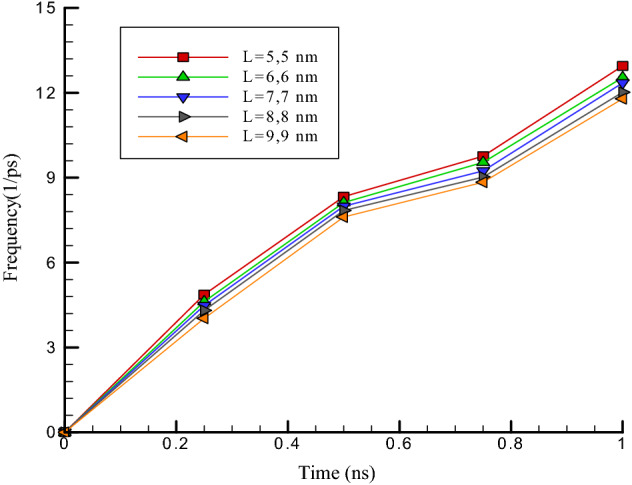
Oscillation frequency changes of carbon nanotubes versus time step with a length of 5 nm in variuos chiralities.

The results show that the amount of interaction energy increases from 55.01 to 58.94 eV by increasing chirality from (5, 5) to (9, 9) (see Table 3). Despite the increase in the amount of interactive energy in these structures, the enhance in chirality leads to a rediuse in oscillation amplitude in the simulated atomic structure (see Fig. 8). So the oscillation frequency in the structures increases (see Fig. 9). This atomic behavior occurs due to increasing the mechanical strength of atomic structures. This behavior follows previous studies in the mechanical manner of carbon nanotubes and indicates the accuracy of the results in our research^25–28^. It should be noted that the value of the frequency can be calculated by storing the position of the atoms in an output file and calculating the periodic motion of the carbon nanotube.

**Table 3:** Highest values of, interaction energy, amplitude and oscillation frequency of carbon nanotubes with a length of 5 nm versus chirality of carbon nanotubes in the last time step of molecular dynamics simulation.

Chirality of carbon nanotubes	Oscillation range (Å)	Oscillation frequency (ps^−1^)	Interactive energy (eV)
(5, 5)	2.97	11.97	55.01
(6, 6)	2.89	12.02	55.22
(7, 7)	2.56	12.35	56.31
(8, 8)	2.2	12.54	58.59
(9, 9)	2.02	12.95	58.94

Finally, the bending stiffness of CNT variation as a function of nanotube chirality was reported. We expected this physical parameter is increasing by chirality increasing in the atomic nanotube. Numerically, by chirality increasing from (5,5) to (9,9), the bending stiffness changes from 0.79 to 0.90 eV, respectively (see Table 4). So we conclude the mechanical strength of simulated structures in MD simulated process increases by C atoms number increasing with chirality parameter.

**Table 4:** Bending Stiffness changes of carbon nanotubes versus chirality of nanotubes.

Chirality of carbon nanotubes (Å)	Oscillation range (Å)
(5,5)	0.79
(6,6)	0.80
(7,7)	0.86
(8,8)	0.088
(9,9)	0.90

**Figure 10:**
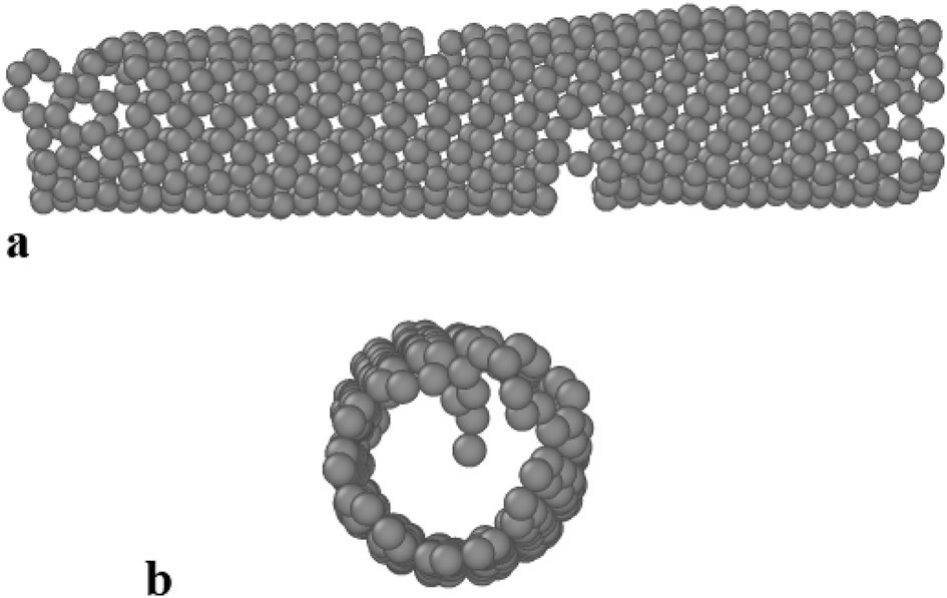
The snapshot of destructed CNT from (**a**) side and (**b**) top views.

**Figure 11:**
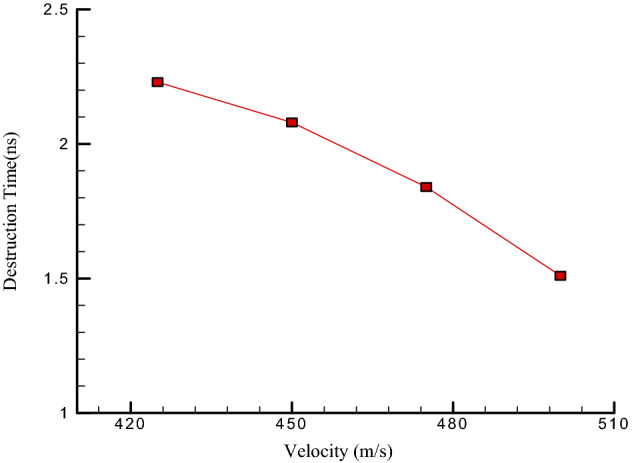
The destruction time of CNT (7,7) with 5 nm length in the vicinity of Ar atoms as a function of fluid velocity.

Finally, we describe the destruction time of CNT (7,7) with a 5 nm length structure as a function of Ar fluid velocity. For this purpose, the velocity of Ar atoms varies from 425 to 500 m/s, and the destruction time of the atomic nanotube is calculated. Atomic arrangement of destructed CNT depicted in Fig. 10 from top and side views. The results show that increasing the velocity of the liquid reduces the degradation time. For example, increasing Ar atoms velocity to 425 m/s, the atomic destruction is detected after t = 2.23 ns (see Fig. 11 and Table 5). Therefore, by increasing the fluid velocity to 500 m/s, the destruction time of CNT decreases to 1.51 ns (See Table 5). The results obtained in this part of the MD study showed the limitation of CNTs performance in nanofluid mixtures, which should be considered in the actual application.

**Table 5:** The destruction time of CNT (7,7) with 5 nm length as a function of Ar atoms velocity.

Ar atoms velocity (m/s)	Destruction time (ns)
400	–
425	2.23
450	2.08
475	1.84
500	1.51

## Conclusion

In this paper, the oscillations and vibrational behavior of a carbon nanotube within a platinum channel in the vicinity of argon flow with a velocity higher than the speed of sound were investigated so that the research’s independent variables were the length of the nanotube and the chirality of the nanotube. The molecular dynamics simulations in balancing structures at initial temperature and pressure showed the stability of atomic structures, which was achieved by combining temperature diagrams and atomic structures’ potential energy. The results of the simulation performed by examining each of the expressed quantities and applying each of the independent variables in this study are as follows:

- This study is balanced in 300 K, and therefore temperature fluctuations are not observed in it, and after 1,000,000 time steps, the atomic structure reaches equilibrium.
- In terms of the simulated system’s potential energy, it can be said that this structure is balanced, and the value is negative and equal to − 22.43 eV.
- With the suitable selection of the interaction between particles and atomic positions in simulated structures, the temperature in these structures converges. As a result, the amplitude of oscillations becomes smaller as the time steps become more and more.
- As the length of carbon nanotubes enhances, the oscillation amplitude’s numerical value decreases to 4.03 nm. Also, the amount of energy converges to 69.75 eV and the frequency in this structure decreases to 10.14 ps^−1^, and.
- By carbon nanotube length increasing from 5 to 25 nm, the stiffness of simulated structures decreases from 0.86 to 0.74 eV, respectively.
- With the increasing chirality of carbon nanotubes, the oscillation amplitude’s numerical value decreases and reaches 2.02 nm. Also, the frequency in this structure decreases to 1.79 ps^−1^, and the number of energy converges to 8.94 eV.
- By chirality increasing from (5,5) to (9,9) the bending stiffness changes from 0.79 to 0.90 eV, respectively.
- The destruction time of the CNT structure decreases from 2.23 to 1.51 ns by Ar atoms velocity enlarging from 425 to 500 m/s, respectively.
